# Evaluation of Large Language Model Performance in Assessing Health Economic Study Quality

**DOI:** 10.36469/001c.145214

**Published:** 2025-10-24

**Authors:** Chen Dun, Cody J. Couperus, Seohu Lee, Robert Barrett, Haeun Lee, Minqi Christelle Xiong, Yiheng Wang, Qingrui Wang, Harold P. Lehmann

**Affiliations:** 1 Biomedical Informatics and Data Science Johns Hopkins University School of Medicine, Baltimore, Maryland; 2 Department of Surgery Johns Hopkins University School of Medicine, Baltimore, Maryland; 3 Critical Care Medicine Johns Hopkins University School of Medicine, Baltimore, Maryland

**Keywords:** artificial intelligence, Large Language Models, CHEERS, publication quality

## Abstract

**Introduction:**

Economic evaluations are essential for informed healthcare decision-making but often face challenges due to inconsistent reporting and methodological complexity. Large Language Models (LLMs) offer a scalable alternative for evaluating adherence to such standards. Building on Hileas, a previously developed tool, this study assesses the accuracy of LLM-generated evaluations compared with human reviewers, aiming to quantify reliability, identify limitations, and advance automated, but assistive quality assessment methods in health economic research.

**Methods:**

In all, 110 peer-reviewed economic evaluation papers were evaluated using the CHEERS checklist through structured LLM prompts and scored by 2 human reviewers on a 0-4 ordinal scale. Interrater agreement and LLM performance were measured using Cohen’s kappa, sensitivity, specificity, and area under the curve. LLM outputs were compared against human consensus ratings, and usability of the review platform was assessed with the System Usability Scale.

**Results:**

Among 2860 item-level evaluations, 25.3% showed disagreement between human reviewers, with generally low interrater reliability (kappa=−0.07 to 0.43). Compared with human consensus, the LLM achieved 72.3% to 94.7% agreement, with areas under the curve up to 0.96 but variable performance across checklist items. At the paper level, LLM-assigned CHEERS scores (median, 17) were consistently lower than human-reviewed scores (median, 18-21).

**Conclusion:**

This study demonstrated an exploratory proof-of-concept application of LLMs to research quality evaluation. Our results suggests that the LLM was generally able to provide well-reasoned evaluations that closely aligned with human assessments, although with some limitations in fully supporting its judgments.

## INTRODUCTION

Economic evaluation plays a critical role in healthcare decision-making by systematically assessing the costs and benefits of medical interventions. Ensuring the rigor and transparency of economic evaluations is essential for their credibility and usability in clinical and policy decision-making. To standardize reporting and improve study quality, guidelines such as the Consolidated Health Economic Evaluation Reporting Standards (CHEERS) have been established.^1^ However, adherence to these frameworks remains inconsistent, and traditional methods of assessing research quality, such as manual review, can be time-consuming, subjective, and prone to bias.^2^ This lack of adherence underscores the need for automated, scalable approaches to systematically evaluate the quality of economic evaluations and decision-analytic models. We aimed at creating an assistive artificial intelligence (AI) tool that works in conjunction with the author as final arbiter of the results (“human in the loop”), rather than autonomous AI that works alone.^3^

Large Language Models (LLMs) offer a transformative solution to this challenge. As the technology advances, publicly available LLMs, such as ChatGPT^4^ and Claude,^5^ are now capable of analyzing a corpus of economic evaluation studies, regardless of size, assessing adherence to established reporting standards and identifying critical gaps that may impact the reliability of findings. Their ability to rapidly extract key components, detect inconsistencies, and provide structured assessments makes them a powerful tool for enhancing research quality and transparency. Building on this potential, we previously developed a web-based interface, Human in the Loop Explainable AI Solutions (Hileas), that utilizes LLMs to review articles and evaluate articles’ compliance with relevant guideline criteria. While this tool introduced a structured approach to assessing guideline adherence, its performance has not yet been formally evaluated. We aimed to systematically assess the performance of LLM-generated evaluations in health economic evaluation research. By comparing LLM-based assessments with human reviews, we seek to quantify the reliability of automated evaluations, identify potential limitations, and refine methodologies for using LLMs in research quality assessment.

## METHODS

### Study Design and Eligibility Criteria

We employed LLMs (ChatGPT-4o, OpenAI) to automatically evaluate the quality of health economic evaluation studies, and human assessment was then conducted to evaluate LLMs’ performance on these evaluations.^4^ To provide a sampling frame for eligible articles, we performed a systematic literature review, identifying economic evaluation papers from PubMed Central Open Access that were licensed under Creative Commons or similar licenses, to prevent nonlicensed papers being shared with public, but proprietary, LLMs.^6,7^ The review included peer-reviewed studies that employ economic modeling or decision analysis in the healthcare domain. To be eligible, studies must have been published in the last 10 years (2015-2024), written in English, and included sufficient methodological details to allow for an assessment of adherence to reporting guidelines. Studies were excluded if they were editorials, commentaries, conference abstracts, protocols, systematic reviews, meta-analyses, or reviews without original analysis. Additionally, papers focused on non-healthcare domains or those with incomplete or inaccessible full texts were excluded. Studies focusing on decision support tools or decision analysis tools rather than decision analysis itself were also excluded. The detailed search criteria are identified in **Supplementary Table S1**. We categorized the identified studies into two groups: health economic evaluations and decision analysis, where decision analysis was a subgroup of economic evaluation.

### Data Extract and Sample Size Calculation

We used PubMed Entrez utilities with the predefined search criteria to identify relevant papers.^8^ Using the PubMed Central (PMC) BioC API (https://www.ncbi.nlm.nih.gov/research/bionlp/APIs/BioC-PMC/), we downloaded the full-text versions of the identified papers. Each paper was assigned a unique identification number and categorized into one of two groups: health economic evaluations and decision analysis studies.

Two separate sample size calculations were conducted. The first was a sample size of the overall cohort (papers) that would result in an adequately large sample that would represent the larger population. Using a proportion test, we set the null hypothesis that the proportion of adherence to reporting guidelines in the sample does not significantly differ from the overall publications. Given an expected proportion of adherence of .50, a margin of error of .10, a 95% confidence level, and a target power of 80%, our sample size calculation suggested that a minimum of 97 papers would be required to ensure representativeness. The second sample size calculation was to assess the number of papers needed to assess agreement between human reviewers and LLM assessments. We calculated the required sample size for Cohen’s κ statistic, which measures interrater reliability while accounting for agreement expected by chance. We assumed an expected κ value of 0.8 (almost perfect agreement) and set 0.2 as the minimal desired detectable difference.^9^ To achieve 80% power as a significant level (α) of 0.05, a minimum of 110 papers was calculated to be needed. We adopted Landis and Koch’s use of the adjectives “low” to “high” agreement.^10^ We decided to select a total of 110 eligible papers in this study to provide additional buffer for variability.

### LLM Assessment Process and Prompting Strategy

To develop the LLM-assessment chatbot, we used the following prompt-engineering strategy. First, a *general* prompt was developed to establish a consistent format for the LLM’s responses. (**Supplementary List**) This general prompt instructed the model to evaluate each item based on defined criteria and to structure its output into 3 key components: a color-coded box indicating whether the guideline item was satisfied (green for satisfied, red for not satisfied, binary coding yes/no). It was also instructed to provide an explanation justifying the assessment and a direct quote extracted from the article that it used to support its evaluation the evaluation.

Second, for each *specific* guideline item, we generated item-specific prompts based on the item description from the CHEERS-defining publication.^11^ To ensure consistency, minimize ambiguity, and allow the model to generate standardized outputs across all items, we directly converted each CHEERS checklist item into a structured yes/no question. For example, the CHEERS item, “Describe the setting and location where data were collected and list the dates of the study,” was reformulated as, “Does the abstract describe the setting and location of data collection, and does it list the study dates?” These targeted prompts were developed through an iterative engineering process, ensuring that the LLM’s evaluation aligned with established reporting standards for health economic evaluation studies. **(Supplementary Table S2).** The LLM was not instructed to review supplementary material of the articles.

### Human Review Process

To validate the accuracy of LLM-generated assessments, each paper was independently reviewed by two human reviewers who were blinded to each other’s evaluation. We implemented a constrained random assignment strategy (Balanced Incomplete Block Design with modifications)^12–16^ to ensure complete coverage and balanced workload: all papers were planned for review, each by 2 different reviewers, and, to minimize burden on them, no individual reviewer assessed more than 26 papers. To minimize clustering and to ensure maximum spread of pairings, each reviewer’s assigned papers were distributed across the remaining 7 reviewers, so that no 2 reviewers shared an excessive number of papers in common. Pseudonyms were provided (eg, *jellybean*) to preserve privacy but enable identification of responses. Reviewers assessed the LLM’s output, its accuracy and its support of its answer, using a 0-4 ordinal scale as the LLM-performance scale, where 0 indicated that the machine gave no answer, 1 indicated a judgment that it gave an inaccurate answer with inadequate support (eg, hallucination or an irrelevance), 2 indicated an inaccurate answer with adequate support, 3 indicated an accurate answer with inadequate support, and 4 indicated an accurate answer with adequate support. When the ratings were binarized (ie, converted to yes or no based on whether the LLM output met the reporting standard), the LLM-performance scale 1 and 2 indicated no, and 3 and 4 indicated yes. The “answer” referred to the color coding (eg, yes or no), and the “support” referred to both explanation and quote. The web interface included 5 clickable score boxes corresponding to the 0-4 scale for each item, allowing reviewers to assign scores based on their assessment of the LLM’s output. All reviewer scores were automatically collected. For the items where the 2 human reviewers assigned different scores, we did not create a consensus rating. Instead, we retained both the lower and higher ratings to assess the range of human judgment and compared this range with the LLM’s assessment. This approach was chosen to capture the natural variability in human interpretation of CHEERS items and to provide a more transparent benchmark for comparison with LLM performance.

At the end of the evaluation, each reviewer was asked to assess system usability. For this survey, we adapted the System Usability Scale (SUS), a long-used and validated 10-question survey that uses a 5-point Likert scale, ranging from 1 (strongly disagree) to 5 (strongly agree).^17^

Human reviewers were informatics students (masters’ or doctoral) and faculty from the Johns Hopkins informatics program. All reviewers participated in a structured training process that included a 1.5-hour session held in a conference room, during which the CHEERS checklist and study protocol were reviewed in detail, then followed by a 4-hour workgroup practice session where reviewers independently applied the criteria to sample abstracts, then discussed discrepancies as a group to promote consistency in interpretation. The training process was held in person in a conference room.

### Statistical Analysis

**Figure 1.**
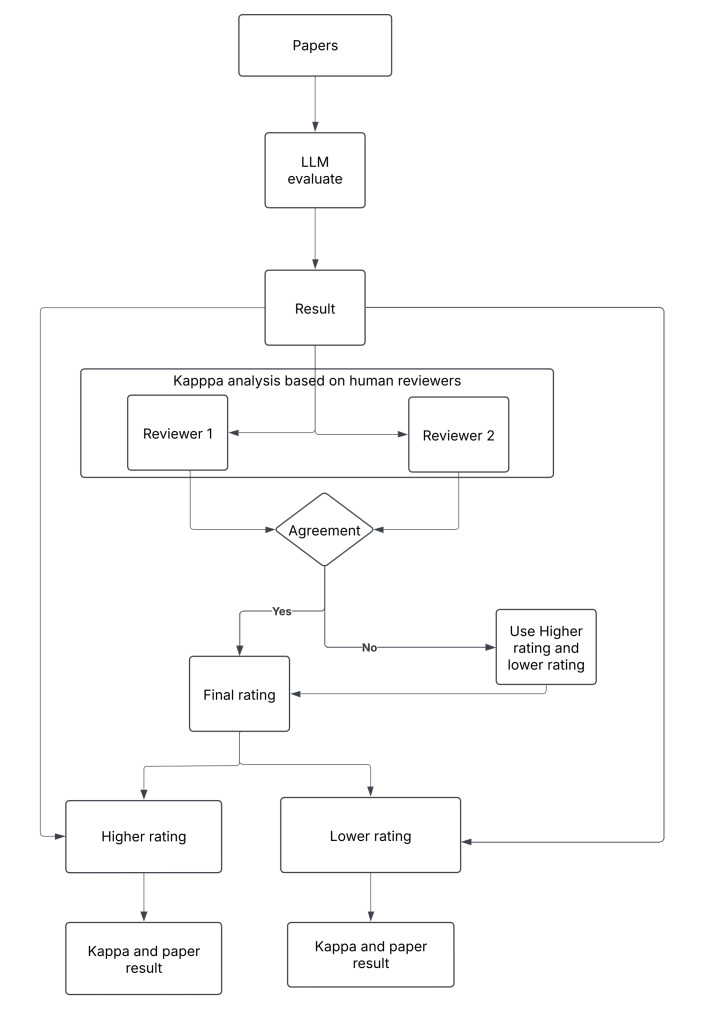
Evaluation Process

The analytic plan is shown in **Figure 1**. We assessed agreement between human expert assessments and interrater agreement across 8 reviewers using Cohen’s κ coefficients. We used a heatmap to illustrate pairwise κ values while further describing the level of agreement for each reviewer in summary statistics. The scores from 2 independent human reviewers were used and generated a rating for each item based on the higher rating and lower rating.

To further evaluate the “accuracy” of the LLM’s assessment, we used human reviewers’ LLM performance scores as the reference standard. Using scores from both reviewers, we calculated the classification performance metrics for each item to assess the LLM’s ability to identify relevant content within the article:

- **Sensitivity**: The proportion of correctly identified components among all true components (true positives), assessing the LLM’s ability to detect guideline-compliant elements
- **Specificity**: The proportion of correctly excluded components among all absent components (true negatives), evaluating the LLM’s ability to correctly identify missing elements
- **Positive predictive value**: The proportion of components identified by the LLM that were correctly classified reflecting the model’s ability to make accurate positive identifications
- **Negative predictive value**: The proportion of components not identified by the LLM that were correctly excluded, reflecting the model’s ability to make accurate negative exclusions
- **Receiver operating characteristic area under the curve (ROC AUC)**: A summary measure of the model’s ability to discriminate between relevant and irrelevant components across all classification thresholds

SUS was reported based on the average score from the reviewers. We also calculated a paper-level score by assigning a binary value to each checklist item (1 = yes, 0 = no) and summing the scores for each article. This calculation was done separately for the LLM-generated and 2 human review results to enable direct comparison. All statistical analyses were conducted using Python (version 3.13.3).

## RESULTS

## Reviewer Rating Comparison

**Figure 2.**
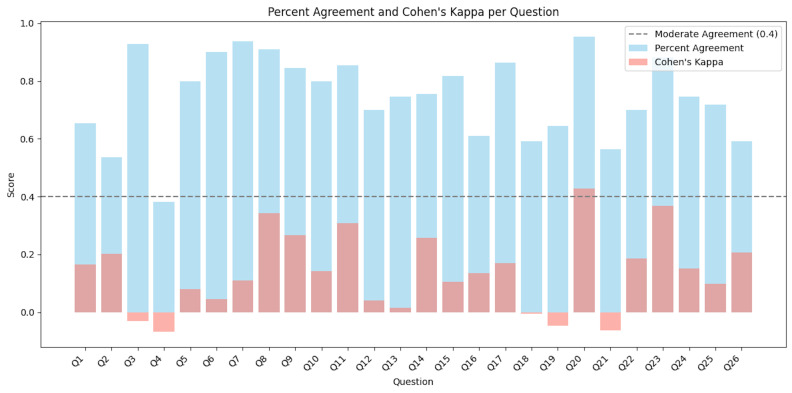
Percent Agreement and Cohen’s Kappa Evaluation for Each Question by 1-4 Rating Scale and Binarized Answer (eg,Yes/No)

The LLM provided answers to all questions. There was a total of 2860 individual-item evaluations (case = 110 papers × 26 CHEERS checklist items) across 8 reviewers. Among the items reviewed, 723 cases (25.3%) had ratings that differed in LLM output assessment. The percent raw agreement across individual items ranged from 28.2% to 95.5%. After adjusting for chance agreement, Cohen’s κ values were generally lower, ranging from −0.07 to 0.43, with several items showing only slight or fair agreement (**Figure 2**). When the ratings were binarized, the percent agreement ranged from 52.7% to 99.1%, and Cohen’s κ values increased, with 30.8% (n = 8) of items achieving moderate to substantial agreement (up to κ = 0.76) (**Supplementary Figure S1).** Even though the LLM and human reviewers sometimes disagreed about whether a checklist item was adequately supported by the manuscript (eg, based on evidence or justification), there were no cases where the LLM completely made up or fabricated information that was not present in the manuscript (ie, no hallucinations).

The final ratings for each item were visualized based on the low rating and higher rating from the reviewers to the LLM (**Supplementary Figure S2**). For the lower rating, across the 110 papers assessed, the distribution of final reviewer ratings (on the LLM-performance scale) varied by CHEERS checklist item (Q1-Q26). Of all item-level assessments, 2067 cases (72.3%) received the highest score of 4. Ratings of 1 (no justification or poor quote support) were given in 339 cases, while intermediate ratings of 2 and 3 were assigned in 302 and 152 cases, respectively. The majority of items demonstrated a high frequency of Rating 4 (indicating a good answer with good support). 11.5% (3/26 questions) had over 100 papers receiving a rating of 4. Conversely, items such as Q2 (abstract), Q4 (analysis plan), Q16 (model), and Q26 (findings and limitations) demonstrated a more mixed distribution, with a higher number of lower ratings. (**Supplementary Table S3**) For the higher rating, of all item-level assessments, 2678 cases (93.6%) received the highest score of 4. Ratings of 1 (no justification or poor quote support) were given in 21 cases (0.7%), while intermediate ratings of 2 and 3 were assigned in 131 and 30 cases, respectively. The majority of questions demonstrated a high frequency of Rating 4; 76.9% (20/26 questions) had over 100 papers receiving a final rating of 4. Like the lower rating, Q2, Q4, Q16, and Q26 still had the greatest range in ratings (**Supplementary Table S4**).

### Interrater Agreement Among Reviewers

Overall, the agreement between pairs of reviewers was low. Mean κ values ranged from 0.067 (reviewer *caramel*) to 0.240 (*lollipop*), indicating poor to slight agreement across most reviewer pairs. Similarly, the median κ values mirrored this trend, with *caramel* showing the lowest median (0.075) and *lollipop* showing the highest (0.226) (**Table 1**).

**Table 1.** Interrater Agreement Among Reviewer Pairs Based on Kappa Statistics

**Reviewer**	**Mean**	**Median**	**Min**	**Max**	**Excellent Agreement, n**	**Moderate Agreement, n**	**Low Agreement, n**
*lollipop*	0.24	0.226	0.015	0.409	0	1	6
*apple pie*	0.214	0.213	0.027	0.425	0	1	6
*milkshake*	0.181	0.108	0.039	0.425	0	1	6
*waffle cone*	0.163	0.133	0.074	0.265	0	0	7
*coconut*	0.155	0.188	-0.049	0.265	0	0	7
*cherry pop*	0.139	0.109	0.027	0.409	0	1	6
*jellybean*	0.094	0.111	-0.049	0.231	0	0	7
*caramel*	0.067	0.075	-0.014	0.121	0	0	7

The range of κ values for each reviewer also highlighted variability in pairwise agreement. For example, *apple pie* and *milkshake* had a maximum pairwise κ of 0.425, suggesting some moderate agreement in isolated comparisons, but their minimum κ was near zero, reflecting inconsistency. No reviewer achieved a pairwise κ in the “excellent” range (≥0.75), and only 3 reviewers (*lollipop, apple pie*, and *cherry pop*) demonstrated a single instance of moderate agreement (κ = 0.40-0.75). The remaining comparisons fell within the low agreement range (κ <0.40), with several reviewers (eg, *waffle cone, coconut, jellybean,* and *caramel*) having no moderate or excellent agreements at all. Notably, *coconut, jellybean*, and *caramel* showed negative minimum κ (as low as −0.049), which indicates worse-than-chance agreement with some peers (**Supplementary Figure S3**).

### Human Results Compared With LLM Result

Given the low agreement between human reviewers, we compared human and LLM assessments within lower ratings of the 2 human reviews and the higher ratings. Using the lower rating, the LLM’s binary classification (yes/no) aligned with the human-reviewed results in 2067 cases, yielding an overall agreement rate of 72.3%. The confusion matrix revealed 1682 true positives, 537 true negatives, 554 false negatives, and 87 false positives. The LLM achieved a sensitivity of 0.752, specificity of 0.861, and an overall ROC AUC of 0.81. Classification performance in model satisfaction with reporting criteria varied across the 26 CHEERS checklist items, with AUC values ranging from 0.46 to 0.97 (**Supplementary Figure S4**). Items such as Q20 (characterizing uncertainty), and Q23 (summary of main results) demonstrated strong discriminative performance, each achieving AUCs greater than 0.90. Notably, Q20 had a sensitivity of 0.95 specificity of 1.00, and an AUC of 0.98, indicating excellent classification ability. In contrast, Q19 (characterizing distributional effects) and Q25 (effect of engagement with patients and others affected by the study) showed weaker performance, with low sensitivity values (0.10-0.12) despite perfect or near-perfect specificity, suggesting the LLM often failed to detect true positives. Interestingly, Q3 (background and objectives) exhibited perfect sensitivity (1.0) but zero or very low specificity, indicating that the model frequently overestimated adherence by marking nearly all instances as positive regardless of ground truth. Detailed performance metrics per question are provided in **Supplementary Table S5**.

Using the high rating, the LLM’s binarized classification (yes/no) aligned with the human-reviewed results in 2708 cases, yielding an overall agreement rate of 94.7%. The confusion matrix revealed 1,761 true positives, 947 true negatives, 144 false negatives, and 8 false positives. The LLM achieved a sensitivity of 0.92, specificity of 0.99, and an overall ROC AUC of 0.96. The AUC values ranged from 0.75 to 1 (**Supplementary Figure S4**). Items such as Q7 (comparators), Q13 (valuation of outcomes), and Q20 (characterizing uncertainty) demonstrated almost perfect discriminative performance, each achieving AUCs greater than or equal to 0.99. In contrast, Q19 (characterizing distributional effects) and Q25 (effect of engagement with patients and others affected by the study) showed weaker performance again, with low sensitivity values (0.50-0.57) despite perfect or near-perfect specificity, suggesting the LLM often failed to detect true positives (**Supplementary Table S6**).

### Paper Quality Score

**Figure 3.**
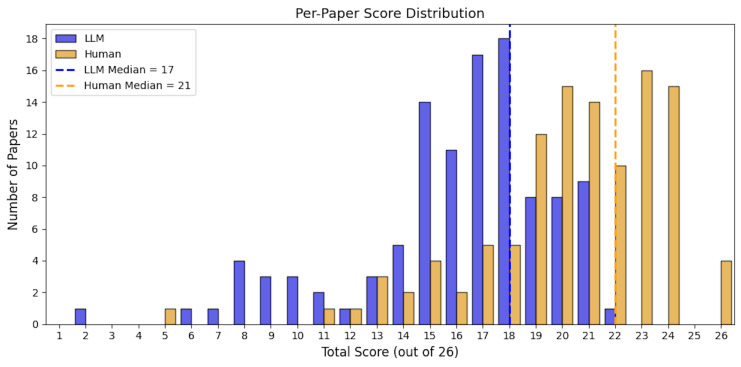
Distribution of Total Paper-Level Quality Scores Assigned by LLM and Human Reviewers. (Lower Rating)

**Figure 4.**
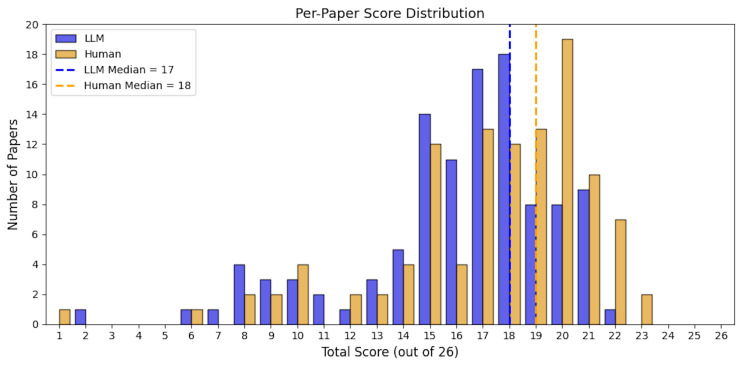
Distribution of Total Paper-Level Quality Scores Assigned by LLM and Human Reviewers (Higher Rating) **Abbreviation**: LLM, Large Language Model.

At the paper level, the average total score assigned by the LLM was 16.1 of 26 (SD = 3.8), compared with an average of 20.3 (SD = 3.5) in the lower human-reviewed LLM-performance scale, and 17.3 (SD = 3.9) for the higher human-reviewed LLM-performance scale. The median score also differed between groups, with LLM median of 17 (IQR: 15, 18) and human median of 21 (IQR: 19, 23) in the lower LLM-performance scale and 18 (IQR: 15, 20) in the higher LLM-performance scale. The LLM-assigned scores ranged from 2 to 22, the human-reviewed scores spanned a wider range, from 5 to 26 for the lower LLM-performance scale and 1 to 23 for higher LLM-performance scale. The LLM’s score distribution was relatively underestimated compared with the human-reviewed results (**Figures 3 and 4**).

### System Usability Scale

Eight participants in the CHEERS evaluation completed the SUS survey, yielding a 100% response rate. The average SUS score was 85.4 (SD = 13.6) out of 100, which is well above the commonly accepted threshold of 68 for acceptable usability and falls within the “excellent” range according to standard benchmarks.

## DISCUSSION

To our knowledge, our study is the first to systematically evaluate research quality in health economic evaluation studies using a web-based interface integrated with an LLM. While LLMs have been explored for helping in some aspects of systematic reviews, their application in automated quality assessment remains underdeveloped.^18^ Our study contributes to the growing field of LLM-assisted research evaluation by providing a structured, methodology-specific approach that integrates guideline-based assessment, human review validation, and a scalable web-based interface.

A key observation of this study was the general lack of agreement among the human reviewers. We suspect that one major cause of this lack of agreement was the variability in experience by the reviewers themselves. However, that variability speaks to the inexactness of the items themselves. While CHEERS was originally designed for human reviewers to simply identify the page on which an item was satisfied, our use was stricter: to decide whether the item was indeed met. A particular source of confusion are items expressed as conjunctions: if either component were missing, it was not clear if the item was satisfied (it met at least part of the requirement) or not (for the logical statement A AND B, if either A or B are false, the conjunction is false). Clearer operational definitions and more standardized training for human reviewers is needed to improve consistency and interpretive alignment. For example, Q8 asks authors to “Describe the setting and location where data were collected and list the dates of the study.” In practice, studies frequently report one component (eg, the study setting) but omit the other (eg, study location). This structure can introduce interpretive ambiguity for both human raters and LLMs, particularly when one element is present but the other is absent or only partially addressed. In our evaluation, such cases frequently led to discrepancies in scoring and contributed to variability in interrater agreement. While we attempted to mitigate this through training and explicit decision rules, the issue reflects a broader challenge in operationalizing complex reporting guidelines. Future work could explore alternative approaches, such as decomposing conjunctive items into discrete sub-criteria, to reduce ambiguity and improve both human and AI reliability in applying the CHEERS framework. Although some disagreement was observed among human reviewers, this may be less critical in real-world use, since Hileas is intended to assist authors in generating initial checklist responses that the authors then can edit. This human-in-the-loop design reduces the need for perfect LLM performance and emphasizes usability and efficiency over automation alone, which is a core feature of our approach.

Our study demonstrated a 72.3% to 94.7% agreement between human reviewer scores and LLM assessments on the CHEERS reporting guidelines, highlighting the potential of LLMs for pre-screening and identifying guideline compliance in health economic evaluations studies. However, these comparisons should be interpreted with caution. The LLM did not review supplemental materials, and checklist items were reformulated into explicit questions for prompting, creating a translation difference from the original CHEERS wording. Human reviewers also represented a source of variability, treated as a random effect, whereas the LLM is a fixed platform, which may partly explain differences in total scores and reliability. These asymmetries suggest that direct human–LLM reliability comparisons capture different sources of bias and should be viewed as exploratory rather than definitive. A generalizability framework could provide a richer way to partition these variance components in future work. This finding is particularly promising, as previous research has shown that LLMs perform well in extracting information from research articles,^19–21^ but their ability to assess study quality has been less consistent.^18,22,23^ While prior studies have explored the use of LLMs in systematic reviews and literature extraction, they often struggle with overgeneralization and difficulty recognizing study-specific biases, leading to discrepancies between LLM-generated quality scores and expert assessments.^19,24^ By systematically comparing LLM assessments to human expert reviews, our study contributes to the broader literature by offering a structured evaluation framework that helps quantify LLM performance. We incorporated a structured 0-4 ordinal human scoring scale (the LLM-performance scale), providing a quantitative benchmark for assessing the accuracy and reliability of LLM-driven evaluations. Additionally, we incorporated direct quotes from the articles, allowing for better verification of the model’s assessments, which enhances transparency and supporting documentation. By refining LLM-based approaches to research quality assessment, this study provided potentials for further improvements in automated prescreening tools, standardizing guideline compliance checks, and integrating LLMs into systematic review workflows.

Our evaluation showed that items such as Q7 (comparators), Q13 (valuation of outcomes), and Q20 (characterizing uncertainty) demonstrated excellent discriminative ability. These items appear to share common structural and semantic features that likely contributed to the LLM performance. First, the prompts were relatively concrete, with clear, unambiguous language that mirrors standard reporting in economic evaluations. For instance, Q7 asked whether the interventions being compared and are described and justified—an element that is almost always stated in the introduction or methods for high-quality studies. Similarly, Q13 asked about how outcomes were valued and in which population, which is the central to any cost-effectiveness or utility-based analysis and typically appear in the methods section. Second, these items reflected core components of economic evaluation frameworks, which are required by reporting standards such as CHEERS.^25^ Because these components are essential for methodological transparency and decision-making, authors are more likely to report them in a structured format or label them in a specific way (eg, “we conducted a probabilistic sensitivity analysis to compare ____”). Another contributing factor is that these items had lower contextual ambiguity. Unlike concepts such as equity consideration (Q19) or stakeholder engagement (Q25), which may be addressed obliquely or scattered across multiple paragraphs, Q7, Q13, and Q20 tended to be self-contained and directly stated, which reduced the need for deep inferential reasoning. This factor likely aligned well with the LLM’s strengths in pattern recognition and surface-level text matching.^26^

In contrast, items such as Q19 (characterizing distributional effects) and Q25 (effect of engagement) consistently showed weaker performance. This indicates that while the LLM rarely incorrectly identified noncompliant studies as compliant (ie, few false positives), it frequently failed to detect actual instances of compliance (ie, many false negatives). These findings suggest the model struggled to identify subtle or less frequently reported content, particularly those involving equity considerations or stakeholder engagement. These topics might be mentioned in more indirect forms within the text. The reverse was also observed: perfect sensitivity but low or zero specificity, meaning the model labeled nearly all studies as adherent regardless of actual compliance. These questions involved broad or loosely interpreted language (eg, Q3 [background and objectives]).

The performance variability may also reflect differences in how often specific elements are reported in practice. Items aligned with reporting standards (eg, study population, comparators, or model description) were detected with higher accuracy, while those tied to newer or less standardized recommendations (eg, equity impacts or patient engagement) were more challenging for the LLM. Nevertheless, these results highlight the importance of contextual clarity in both prompt engineering and evaluation criteria. Future iterations of this tool could benefit from fine-tuning the prompts for lower-performing items and incorporating examples during model training that emphasize diverse forms of compliance reporting.

Another important finding of our study was that the LLM tended to underestimate CHEERS scores compared with human reviewers. This conservative bias has several implications. On the one hand, underestimation reduces the risk of overstating study quality, which may be preferable in quality appraisal where false positives could mislead evidence synthesis. On the other hand, consistent underestimation could undervalue studies that are appropriately reported, potentially discouraging their inclusion or creating unnecessary burden for human reviewers to “correct” the AI assessment. This suggests that LLM-based appraisal tools should be positioned as assistive rather than autonomous, with human oversight to balance the risks of over- and underestimation.

A key strength of this study is the utilization of an interactive web-based interface that integrates LLM assessments with human review. Unlike previous LLM applications that relied on open-ended responses with little structure, our approach standardized LLM-generated outputs into 3 key components: a color-coded compliance indicator, a text-based explanation, and a direct quote from the article to support the evaluation. This structured format improves interpretability and transparency, allowing human reviewers to rapidly validate and refine the LLM’s assessments. The interface also enables users to upload multiple documents simultaneously, addressing limitations in existing tools that require manual, one-at-a-time analysis. Furthermore, the improved version of our interface allows researchers to directly link search queries to PubMed Central, streamlining the process of retrieving and evaluating relevant studies. This improvement also creates an opportunity to extend our study to other reporting guidelines, such as STROBE or PRISMA, which are more commonly used in the observational and systematic review studies.

To improve the accuracy and consistency of LLM-generated evaluations, we employed a multiple interactive agent approach for prompting. Instead of relying on a single model instance to generate responses to multiple questions at once, we designed our system to use separate agents for each individual question. Specifically, we first used a general prompt to ensure that all LLM responses followed a structured output format. Then, for each specific evaluation criterion, we assigned a dedicated agent that focused solely on answering that particular question. This question-specific agent approach ensured that each query was addressed independently, preventing interference from unrelated contextual elements. Prior research has identified several limitations associated with prompting LLMs to answer multiple questions in a single input, including variable accuracy, context dependence leading to incomplete or misleading responses, multiple-choice bias, and sensitivity to phrasing.^27–29^ When multiple complex questions are embedded within the same prompt, LLMs may prioritize certain aspects while neglecting others, introduce inconsistencies, or generate answers influenced by unintended contextual cues. By deploying multiple independent agents, our approach mitigates these issues by ensuring that each query is processed in isolation, leading to more precise, contextually relevant, and unbiased responses. This structured agent-based prompting approach represents a key methodological improvement over prior single-prompt evaluation. It significantly enhances the reliability of LLM-assisted research assessments and demonstrates the potential for fine-grained, multi-agent LLM strategies in systematic research evaluation tasks.

### Limitations

Our study had several limitations. One major challenge in using LLMs to assess research quality is that they can be biased and tend to focus only on obvious or superficial details instead of deeply understanding the study’s methodology. Studies evaluating ChatGPT in systematic reviews have found that LLMs tend to provide overly optimistic risk-of-bias assessments, often underestimating critical methodological flaws.^22,30,31^ This means human experts are still needed to review and interpret the results because LLMs might overlook details that depend on the study’s specific context. Our interrater agreement among reviewers suggested substantial variability in reviewer interpretation or application of the evaluation criteria, warranting further training, clearer guidelines, consensus-building procedures, and involvement of expert raters beyond students/faculty. While LLMs can help make assessments more consistent, they are not yet able to think like experts who have deep knowledge of the field. Future research should explore methods for fine-tuning LLMs on domain-specific datasets, improving their ability to detect complex methodological flaws in different studies. In this study, we translated CHEERS checklist items into structured yes/no questions to create clear and standardized prompts for the LLM. While this pragmatic approach ensured reproducibility and minimized ambiguity, it does not align with psychometric standards for translation and validation, such as forward-backward translation, expert review, or cognitive debriefing. As a result, our prompts reflect face-valid engineering choices rather than formally validated instruments. Future work could build on this by incorporating more rigorous translation processes to ensure conceptual equivalence and improve generalizability across contexts. Another key consideration is the comparability of LLM performance across different study types. In this study, we conducted a comparative analysis between economic evaluations and decision analysis studies to assess whether LLM accuracy varied between these two groups. We identified potential differences in sensitivity, specificity, and agreement metrics across study types. These findings offer important insights into the generalizability of LLM-based assessment metrics, which might be potentially helpful to determine whether distinct methodological approaches are needed for different types of research studies. Meanwhile, we evaluated only a single LLM (ChatGPT-4o), which may limit the generalizability of our findings to other LLMs with different architectures, training data, or performance characteristics. In future studies, we plan to extend this work by systematically comparing the performance of multiple LLMs, which will allow us to assess whether our findings hold across models and to identify model-specific strengths or limitations.

The next steps will be to broaden the evaluation framework across more methodology checklists. As we evaluate LLM performance across these checklists, we work toward a general approach to prompt engineering, to the degree possible, and toward mitigation of any biases our evaluations are able to surface.

## CONCLUSION

Our study demonstrated an exploratory proof-of-concept application of LLMs to research quality evaluation. Our results suggested that an LLM has the potential to automate quality assessment for economic evaluations with high overall accuracy and no hallucinations, but with notable specific blind spots in detecting nuanced or infrequently reported elements. This LLM-based model, especially when used as assistive rather than autonomous AI, offers a potential approach to streamlining the review process, improving both efficiency and accuracy, and ultimately enhancing the reliability of evidence synthesis in research.

### Acknowledgments

We acknowledge the use of OpenAI’s large language model (ChatGPT) in the development and evaluation of the Hileas system. The LLM was instrumental in generating assessments based on structured reporting checklists and served as the foundation for this proof-of-concept study.

## Supplementary Material

Online Supplementary Material
