# Supplementary material for: Evaluation of Large Language Model Performance in Assessing Health Economic Study Quality
Source: J Health Econ Outcomes Res. 2025 Oct 24;12(2):154–62. doi: 10.36469/001c.145214 (PMC12554303; doi:10.36469/001c.145214)
Supplement: Online Supplementary Material [file jheor_2025_12_2_145214_307667.pdf]

Journal of Health Economics and Outcomes Research

Online Supplementary Material

Evaluation of Large Language Model Performance in Assessing Health Economic Study Quality. *JHEOR*. 2025;12(2):154-162. doi:10.36469/jheor.2025.145214

Table S1: Search Criteria

List: General Prompt Used to Establish a Consistent Format for the LLM Response

Table S2: CHEERS Reporting Guideline and Targeted Prompts for Each Item

Figure S1: Percent Agreement and Cohen's Kappa Evaluation for Each Question by Binarized Answer (eg, Yes/No)

Figure S2: Low and High Rating for 110 Articles for Each Question: (A) Lower Rating; (B) Higher Rating

Table S3: Rating Distributions for Each CHEERS Item Based on Human Review (Lower Rating Groups)

Table S4: Rating Distributions for Each CHEERS Item Based on Human Review (Higher Rating Groups)

Figure S3: Cohen's Kappa Metric Between Independent Reviewers

Figure S4: Comparison of LLM Performance Using Lower vs Higher Human Rating Thresholds for Binary Classification Across CHEERS Items

Table S5: Confusion Matrix and Performance Metrics Using Lower Rating

Table S6: Confusion Matrix and Performance Metrics Using Higher Rating

This supplementary material has been provided by the authors to give readers additional information about

This is an open-access article distributed under the terms of the Creative Commons Attribution 4.0 International License (CCBY-4.0). View this license's legal deed at http://creativecommons.org/licenses/by/4.0 and legal code at http://creativecommons.org/licenses/by/4.0/legalcode for more information.

Table S1. Search Criteria

Search No.	Query	Sort By	Filters	Search Details	Results	Time
9	#8 and #2			("decision tree"[Title/Abstract] OR "decision analysis"[Title/Abstract] OR "decision model"[Title/Abstract] OR "Markov model"[Title/Abstract] OR "simulation model"[Title/Abstract]) AND ((("Cost-Benefit Analysis"[MeSH Terms] OR "Cost-Benefit Analysis"[Title] OR "cost-benefit"[Title] OR "Cost-Effectiveness Analysis"[MeSH Terms] OR "Economic Evaluation"[Title] OR "Economic Evaluations"[Title] OR "Health Economic Evaluation"[Title] OR "cost minimization"[Title] OR "cost utility"[Title] OR "cost offsets"[Title] OR "cost offset"[Title] OR "cost analysis"[Title] OR "cost analyses"[Title] OR "distributional cost-effectiveness"[Title/Abstract] OR "equity analysis"[Title/Abstract] OR "extended cost-effectiveness"[Title/Abstract]) AND "english"[Language] AND 2015/01/01:2024/12/31[Date - Publication] AND "pmc open access"[Filter]) NOT ("Review"[Publication Type] OR "Systematic Reviews as Topic"[MeSH Terms] OR "systematic"[Filter] OR "Comment"[Publication Type] OR "Editorial"[Publication Type] OR "Letter"[Publication Type] OR "study protocol"[Title] OR "study protocols"[Title] OR "protocol"[Title]))	1733	11:46:57
12	"decision tree"[tiab] OR "decision analysis"[tiab] OR "decision model"[tiab] OR			"decision tree"[Title/Abstract] OR "decision analysis"[Title/Abstract] OR "decision model"[Title/Abstract] OR "Markov	44,995	11:46:18

Search No.	Query	Sort By	Filters	Search Details	Results	Time
	"Markov model"[tiab] OR "simulation model"[tiab]			model"[Title/Abstract] OR "simulation model"[Title/Abstract]		
8	"decision tree"[Title/Abstract] OR "decision analysis"[Title/Abstract] OR "decision model"[Title/Abstract] OR "Markov model"[Title/Abstract] OR "simulation model"[Title/Abstract]			"decision tree"[Title/Abstract] OR "decision analysis"[Title/Abstract] OR "decision model"[Title/Abstract] OR "Markov model"[Title/Abstract] OR "simulation model"[Title/Abstract]	44,995	11:43:02
7	#2 AND #6			((("Cost-Benefit Analysis"[MeSH Terms] OR "Cost-Benefit Analysis"[Title] OR "cost-benefit"[Title] OR "Cost-Effectiveness Analysis"[MeSH Terms] OR "Economic Evaluation"[Title] OR "Economic Evaluations"[Title] OR "Health Economic Evaluation"[Title] OR "cost minimization"[Title] OR "cost utility"[Title] OR "cost offsets"[Title] OR "cost offset"[Title] OR "cost analysis"[Title] OR "cost analyses"[Title] OR "distributional cost-effectiveness"[Title/Abstract] OR "equity analysis"[Title/Abstract] OR "extended cost-effectiveness"[Title/Abstract]) AND "english"[Language] AND 2015/01/01:2024/12/31[Date - Publication] AND "pmc open access"[Filter]) NOT ("Review"[Publication Type] OR "Systematic Reviews as Topic"[MeSH Terms] OR "systematic"[Filter] OR "Comment"[Publication Type] OR "Editorial"[Publication Type] OR "Letter"[Publication Type] OR "study protocol"[Title] OR "study protocols"[Title] OR "protocol"[Title])) AND ("decision tree"[Title/Abstract] OR "decision	656	11:22:01

Search No.	Query	Sort By	Filters	Search Details	Results	Time
				analysis"[Title/Abstract] OR "decision model"[Title/Abstract])		
6	"decision tree"[tiab] OR "decision analysis"[tiab] OR "decision model"[tiab]			"decision tree"[Title/Abstract] OR "decision analysis"[Title/Abstract] OR "decision model"[Title/Abstract]	23,836	11:21:35
5	#3 AND #2			("decision tree"[All Fields] OR "decision analysis"[All Fields] OR "decision model"[All Fields]) AND ((("Cost-Benefit Analysis"[MeSH Terms] OR "Cost-Benefit Analysis"[Title] OR "cost-benefit"[Title] OR "Cost-Effectiveness Analysis"[MeSH Terms] OR "Economic Evaluation"[Title] OR "Economic Evaluations"[Title] OR "Health Economic Evaluation"[Title] OR "cost minimization"[Title] OR "cost utility"[Title] OR "cost offsets"[Title] OR "cost offset"[Title] OR "cost analysis"[Title] OR "cost analyses"[Title] OR "distributional cost-effectiveness"[Title/Abstract] OR "equity analysis"[Title/Abstract] OR "extended cost-effectiveness"[Title/Abstract]) AND "english"[Language] AND 2015/01/01:2024/12/31[Date - Publication] AND "pmc open access"[Filter]) NOT ("Review"[Publication Type] OR "Systematic Reviews as Topic"[MeSH Terms] OR "systematic"[Filter] OR "Comment"[Publication Type] OR "Editorial"[Publication Type] OR "Letter"[Publication Type] OR "study protocol"[Title] OR "study protocols"[Title] OR "protocol"[Title]))	660	11:20:22

Search No.	Query	Sort By	Filters	Search Details	Results	Time
3	"decision tree"[All Fields] OR "decision analysis"[All Fields] OR "decision model"[All Fields]			"decision tree"[All Fields] OR "decision analysis"[All Fields] OR "decision model"[All Fields]	23,997	11:19:07
2	("Cost-Benefit Analysis"[Mesh] OR "Cost-Benefit Analysis"[ti] OR "cost-benefit"[ti] OR "Cost-Effectiveness Analysis"[Mesh] OR "Economic Evaluation"[ti] OR "Economic Evaluations"[ti] OR "Health Economic Evaluation"[ti] OR "cost minimization"[ti] OR "cost utility"[ti] OR "cost offsets"[ti] OR "cost offset"[ti] OR "cost analysis"[ti] OR "cost analyses"[ti] OR "distributional cost-effectiveness"[tiab] OR "equity analysis"[tiab] OR "extended cost-effectiveness"[tiab]) AND english[lang] AND ("2015/01/01"[PDAT] : "2024/12/31"[PDAT]) AND (pmc open access[filter]) NOT (Review[pt] OR "Systematic Reviews as Topic"[Mesh] OR systematic[sb] OR Comment[pt] OR Editorial[pt] OR Letter[pt] OR "study protocol"[ti] OR "study protocols"[ti] OR protocol[ti])			(("Cost-Benefit Analysis"[MeSH Terms] OR "Cost-Benefit Analysis"[Title] OR "cost-benefit"[Title] OR "Cost-Effectiveness Analysis"[MeSH Terms] OR "Economic Evaluation"[Title] OR "Economic Evaluations"[Title] OR "Health Economic Evaluation"[Title] OR "cost minimization"[Title] OR "cost utility"[Title] OR "cost offsets"[Title] OR "cost offset"[Title] OR "cost analysis"[Title] OR "cost analyses"[Title] OR "distributional cost-effectiveness"[Title/Abstract] OR "equity analysis"[Title/Abstract] OR "extended cost-effectiveness"[Title/Abstract]) AND "english"[Language] AND 2015/01/01:2024/12/31[Date - Publication] AND "pmc open access"[Filter]) NOT ("Review"[Publication Type] OR "Systematic Reviews as Topic"[MeSH Terms] OR "systematic"[Filter] OR "Comment"[Publication Type] OR "Editorial"[Publication Type] OR "Letter"[Publication Type] OR "study protocol"[Title] OR "study protocols"[Title] OR "protocol"[Title])	8,944	11:16:45

List. General Prompt Used to Establish a Consistent Format for the LLM Response

"You will be provided with a research article manuscript. Your task is to answer a specific question about the article based on the evidence presented in the text.

For the question, follow these steps:

- Indicator: Provide a clear and concise answer of either "true" or "false" based on the evidence in the manuscript.
- Explanation: Write a brief summary (2-3 sentences) explaining why you chose "true" or "false." Focus on the key evidence or reasoning from the manuscript.
- Supporting Evidence:

If the answer is "true," provide direct quotes from the manuscript that support your answer ensuring they are relevant and clearly tied to the question.

If the answer is "false," explain why the manuscript does not support the question, and also provide quotes support your explanation.

Guidelines

- Ensure the quotes are accurate and directly relevant to the question.
- Do not truncate quotes with ellipses.

Example format for response:

<answer>
True/False
</answer>

<explanation>
[Brief explanation of why the answer is true or false.]
</explanation>

<supporting_evidence>
- [Quote 1]
- [Quote 2]
</supporting_evidence>

Example response:

<answer>
True
</answer>

<explanation>
The article clearly describes how outcomes were measured in the methods section. It specifies key endpoints such as life-years gained, quality-adjusted life-years (QALYs), and incremental cost-effectiveness ratios (ICERs) and details the modeling approach used to capture both the benefits (e.g., fracture risk reduction) and the harms (e.g., costs and quality of life reductions associated with fractures).
</explanation>

<supporting_evidence> [should be DIRECT quotes from the provided manuscript]
"The primary endpoints of the model were life-years gained, quality-adjusted life-years (QALYs), and incremental cost-effectiveness ratios for denosumab against the comparators."

"Patients who sustained fractures incurred fracture-specific costs and reductions in health utilities."

</supporting_evidence>

""""

Table S2. CHEERS Reporting Guideline and Targeted Prompts for Each Item

**Item No.**	**Item Name**	**Guidance from the instrument …**	***… translated into prompt***
**1**	Title	Identify the study as an economic evaluation and specify the interventions being compared.	*Does the title clearly identify the study as an economic evaluation and specify the interventions being compared?*
**2**	Abstract	Provide a structured summary that highlights context, key methods, results and alternative analyses.	*Does the abstract provide a structured summary that includes the context, key methods, results, and alternative analyses?*
**3**	Background and objectives	Give the context for the study, the study question and its practical relevance for decision making in policy or practice.	*Does the introduction provide the context for the study, the study question, and its practical relevance for decision-making in policy or practice?*
**4**	Health economic analysis plan	Indicate whether a health economic analysis plan was developed and where available.	*Was a health economic analysis plan developed, and if so, where is it available?*
**5**	Study population	Describe characteristics of the study population (such as age range, demographics, socioeconomic, or clinical characteristics).	*Are the characteristics of the study population (e.g., age range, demographics, socioeconomic, or clinical characteristics) described?*
**6**	Setting and location	Provide relevant contextual information that may influence findings.	*Is relevant contextual information provided that may influence the findings of the study?*
**7**	Comparators	Describe the interventions or strategies being compared and why chosen.	*Are the interventions or strategies being compared described, along with the rationale for their selection?*
**8**	Perspective	State the perspective(s) adopted by the study and why chosen.	*What perspective(s) were adopted by the study, and why were they chosen?*
**9**	Time horizon	State the time horizon for the study and why appropriate.	*What is the time horizon for the study, and why is it appropriate?*
**10**	Discount rate	Report the discount rate(s) and reason chosen.	*What discount rate(s) were used, and what was the rationale for choosing them?*
**11**	Selection of outcomes	Describe what outcomes were used as the measure(s) of benefit(s) and harm(s).	*What outcomes were used as measures of benefit and harm?*
**12**	Measurement of outcomes	Describe how outcomes used to capture benefit(s) and harm(s) were measured.	*How were the outcomes used to capture benefits and harms measured?*
**13**	Valuation of outcomes	Describe the population and methods used to measure and value outcomes.	*What population and methods were used to measure and value the outcomes?*
**14**	Measurement and valuation of	Describe how costs were valued.	*How were the costs valued in the study?*

Item No.	Item Name	Guidance from the instrument …	*… translated into prompt*
	resources and costs		
**15**	Currency, price date, and conversion	Report the dates of the estimated resource quantities and unit costs, plus the currency and year of conversion.	*What are the dates of the estimated resource quantities and unit costs, and what currency and year were used for conversion?*
**16**	Rationale and description of model	If modelling is used, describe in detail and why used. Report if the model is publicly available and where it can be accessed.	*If a model was used, was it described in detail, including the rationale for its use? Is the model publicly available, and where can it be accessed?*
**17**	Analytics and assumptions	Describe any methods for analysing or statistically transforming data, any extrapolation methods, and approaches for validating any model used.	*What methods were used for analyzing or statistically transforming data, extrapolation, and validating any models used?*
**18**	Characterizing heterogeneity	Describe any methods used for estimating how the results of the study vary for sub-groups.	*What methods were used to estimate how the results vary for different sub-groups?*
**19**	Characterizing distributional effects	Describe how impacts are distributed across different individuals or adjustments made to reflect priority populations.	*How were the impacts distributed across different individuals, and were adjustments made to reflect priority populations?*
**20**	Characterizing uncertainty	Describe methods to characterize any sources of uncertainty in the analysis.	*What methods were used to characterize sources of uncertainty in the analysis?*
**21**	Approach to engagement with patients and others affected by the study	Describe any approaches to engage patients or service recipients, the general public, communities, or stakeholders in the design of the study.	*Were patients, service recipients, the general public, communities, or stakeholders engaged in the design of the study? If so, how?*
**22**	Study parameters	Report all analytic inputs including uncertainty or distributional assumptions.	*Were all analytic inputs (e.g., values, ranges, references) reported, including uncertainty or distributional assumptions?*
**23**	Summary of main results	Report the mean values for the main categories of costs and outcomes of interest and summarise them in the most appropriate overall measure.	*Were the mean values for the main categories of costs and outcomes reported, and were they summarized in the most appropriate overall measure?*
**24**	Effect of uncertainty	Describe how uncertainty about analytic judgments, inputs, or projections affect findings. Report the effect of choice of discount rate and time horizon, if applicable.	*How did uncertainty about analytic judgments, inputs, or projections affect the findings? Was the effect of the choice of discount rate and time horizon reported, if applicable?*
**25**	Effect of engagement with patients and	Report on any difference patient/service recipient, general public, community, or stakeholder	*Did patient, service recipient, general public, community, or stakeholder involvement make*

Item No.	Item Name	Guidance from the instrument …	*… translated into prompt*
	others affected by the study	involvement made to the approach or findings of the study.	*a difference to the approach or findings of the study?*
**26**	Study findings, limitations, generalizability, and current knowledge	Report key findings, limitations, ethical or equity considerations not captured, and how these could impact patients, policy, or practice.	*Were the key findings, limitations, ethical or equity considerations, and their potential impact on patients, policy, or practice reported?*

Figure S1. Percent Agreement and Cohen's Kappa Evaluation for Each Question by Binarized Answer (eg, Yes/No)

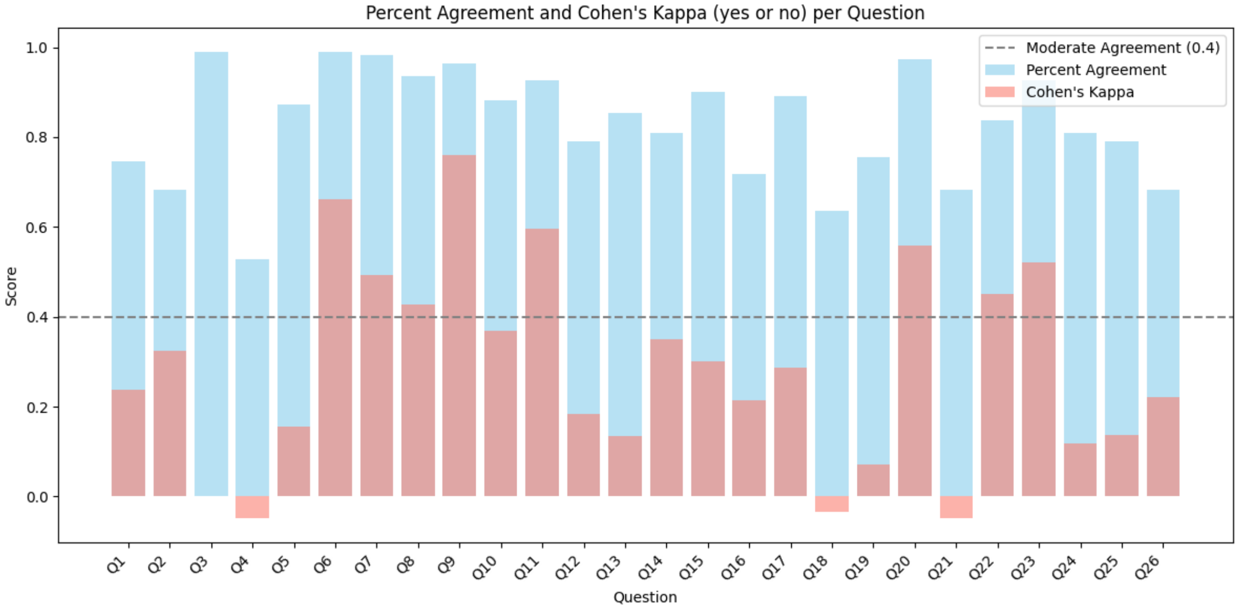

Figure S2. Low and High Rating for 110 Articles for Each Question: (A) Lower Rating; (B) Higher Rating

(A)

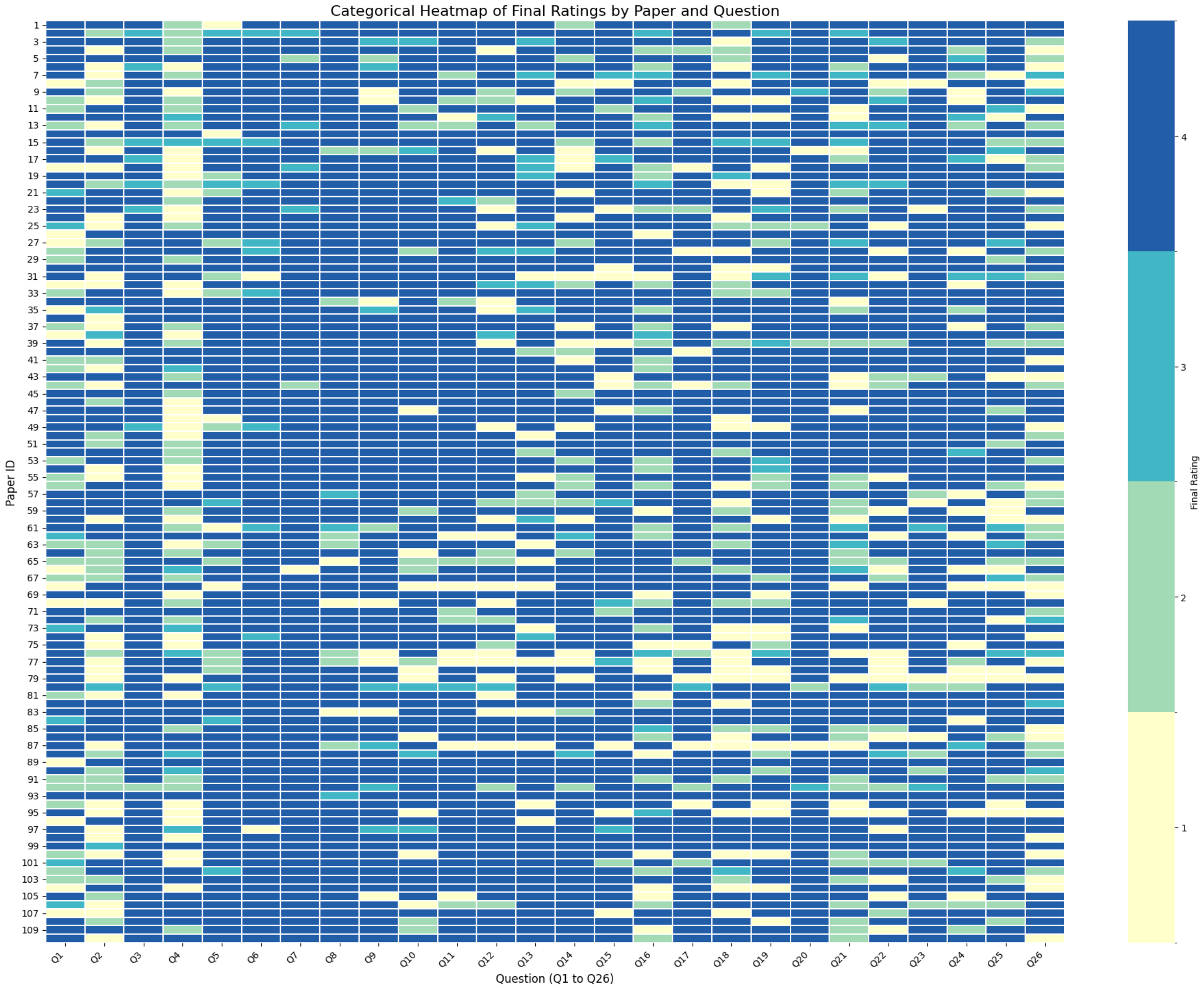

(B)

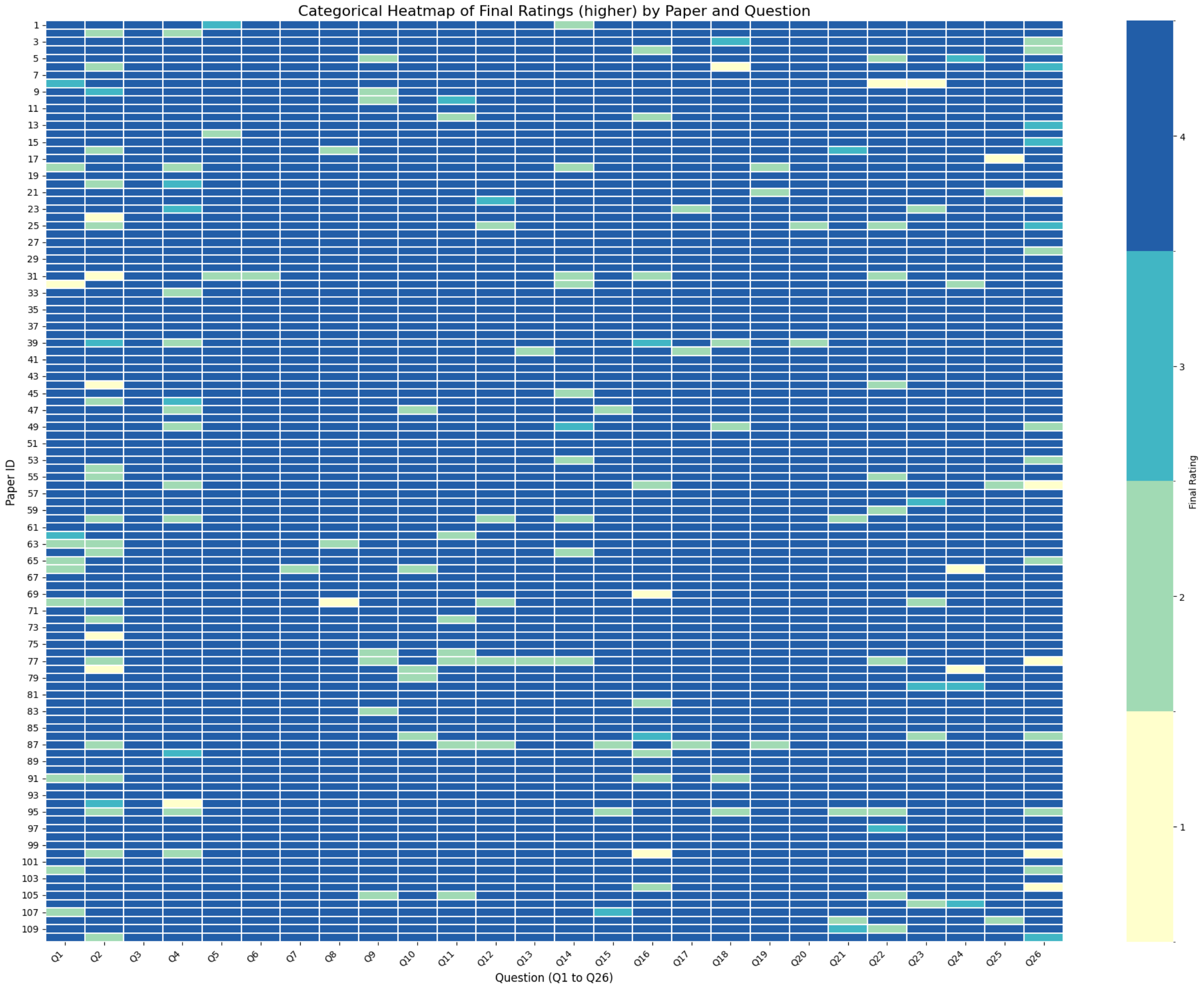

Categorical Heatmap of Final Ratings (higher) by Paper and Question
Paper ID
Question (Q1 to Q26)
Final Rating

Table S3. Rating Distributions for Each CHEERS Item Based on Human Review (Lower Rating Groups)

Question	Rating 1	Rating 2	Rating 3	Rating 4	Total
Q1	14	23	7	66	110
Q2	35	24	4	47	110
Q3	0	1	7	102	110
Q4	34	29	9	38	110
Q5	5	11	7	87	110
Q6	2	0	9	99	110
Q7	1	2	4	103	110
Q8	3	7	3	97	110
Q9	8	3	7	92	110
Q10	9	9	5	87	110
Q11	7	8	2	93	110
Q12	18	10	5	77	110
Q13	13	5	11	81	110
Q14	15	15	2	78	110
Q15	11	3	6	90	110
Q16	13	28	9	60	110
Q17	8	7	1	94	110
Q18	28	17	3	62	110
Q19	19	11	9	71	110
Q20	2	3	2	103	110
Q21	15	23	11	61	110
Q22	19	10	6	75	110
Q23	6	7	2	95	110
Q24	16	8	7	79	110
Q25	14	13	8	75	110
Q26	24	25	6	55	110

Table S4. Rating Distributions for Each CHEERS Item Based on Human Review (Higher Rating Groups)

Question	Rating 1	Rating 2	Rating 3	Rating 4	Total
Q1	1	8	2	99	110
Q2	5	19	3	83	110
Q3	0	0	0	110	110
Q4	1	10	4	95	110
Q5	0	2	1	107	110
Q6	0	1	0	109	110
Q7	0	1	0	109	110
Q8	1	2	0	107	110
Q9	0	7	0	103	110
Q10	0	5	0	105	110
Q11	0	7	1	102	110
Q12	0	5	1	104	110
Q13	0	2	0	108	110
Q14	0	9	1	100	110
Q15	0	3	1	106	110
Q16	2	8	2	98	110
Q17	0	3	0	107	110
Q18	1	4	1	104	110
Q19	0	3	0	107	110
Q20	0	2	0	108	110
Q21	0	3	2	105	110
Q22	1	10	1	98	110
Q23	1	4	2	103	110
Q24	2	1	3	104	110
Q25	1	3	0	106	110
Q26	5	9	5	91	110

Figure S3. Cohen's Kappa Metric Between Independent Reviewers

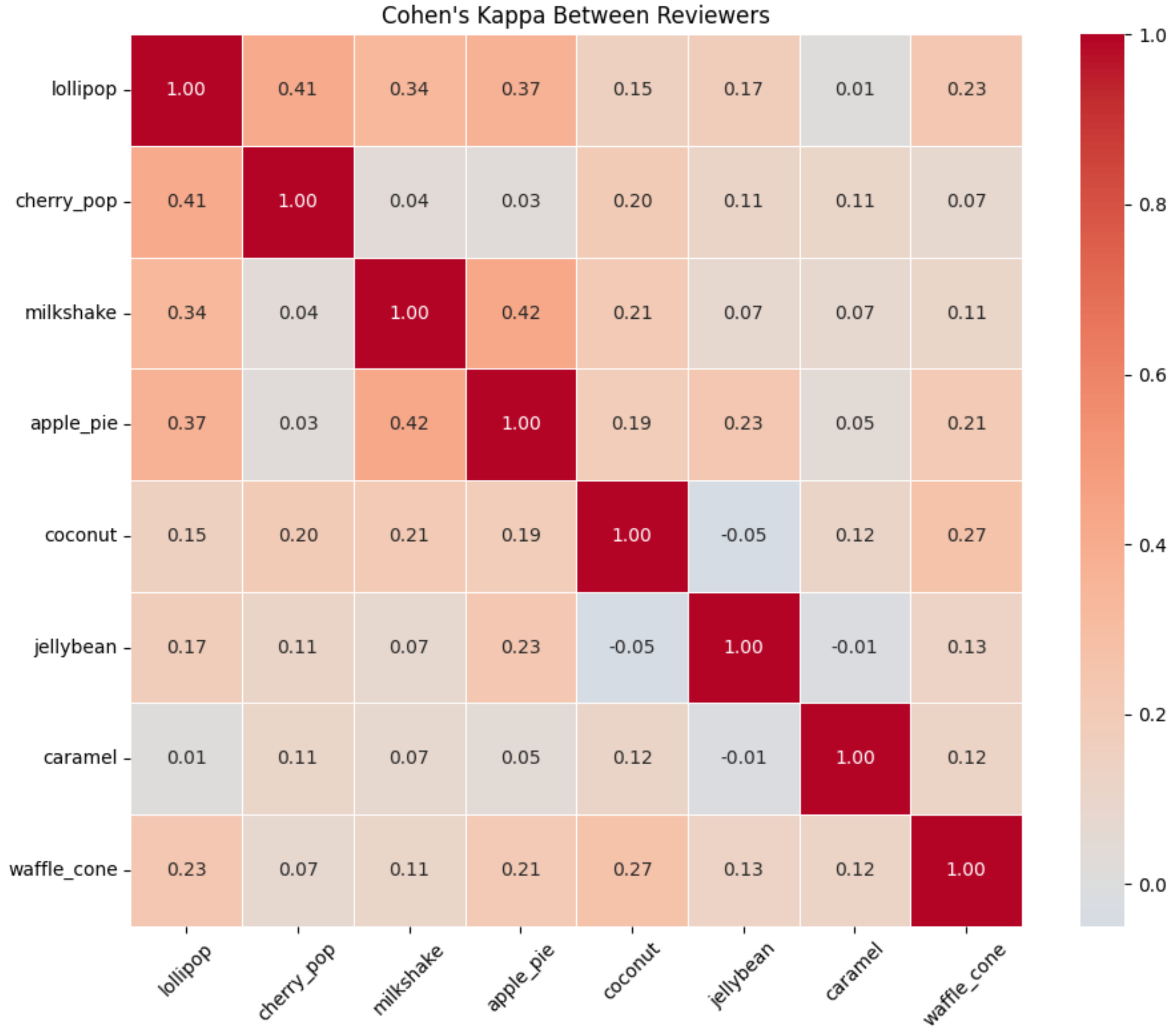

Figure S4. Comparison of LLM Performance Using Lower vs Higher Human Rating Thresholds for Binary Classification Across CHEERS Items

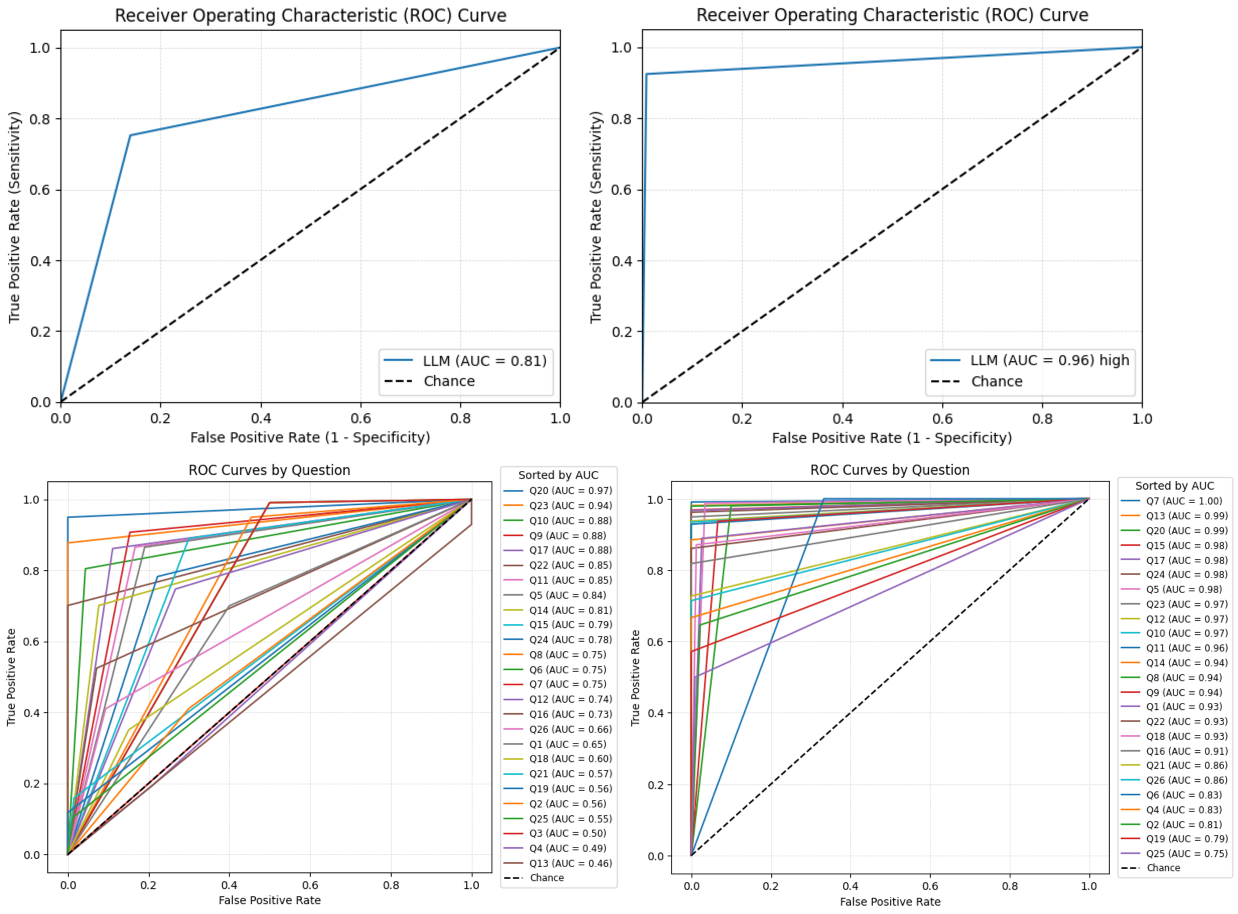

Upper Left, Overall - Lower Rating
Upper Right, Item Level - Lower Rating
Lower Left, Overall - Higher Rating
Lower Right, Item Level - Higher Rating

Table S5. Confusion Matrix and Performance Metrics Using Lower Rating

Question	True Negative	False Positive	False Negative	True Positive	Sensitivity	Specificity	AUC
Q1	24	16	21	49	0.7	0.6	0.65
Q2	14	6	53	37	0.411	0.7	0.556
Q3	0	1	0	109	1	0	0.5
Q4	34	9	54	13	0.194	0.791	0.492
Q5	17	4	12	77	0.865	0.81	0.837
Q6	1	1	1	107	0.991	0.5	0.745
Q7	2	2	1	105	0.991	0.5	0.745
Q8	6	5	5	94	0.949	0.545	0.747
Q9	11	2	9	88	0.907	0.846	0.877
Q10	22	1	17	70	0.805	0.957	0.881
Q11	5	1	14	90	0.865	0.833	0.849
Q12	11	4	24	71	0.747	0.733	0.74
Q13	0	11	7	92	0.929	0	0.465
Q14	12	1	29	68	0.701	0.923	0.812
Q15	7	3	11	89	0.89	0.7	0.795
Q16	26	2	39	43	0.524	0.929	0.726
Q17	8	1	14	87	0.861	0.889	0.875
Q18	45	8	37	20	0.351	0.849	0.6
Q19	76	0	30	4	0.118	1	0.559
Q20	11	0	5	94	0.949	1	0.975
Q21	65	1	37	7	0.159	0.985	0.572
Q22	13	0	29	68	0.701	1	0.851
Q23	4	0	13	93	0.877	1	0.939
Q24	14	4	20	72	0.783	0.778	0.78
Q25	80	1	26	3	0.103	0.988	0.546
Q26	29	3	46	32	0.41	0.906	0.658

Table S6. Confusion Matrix and Performance Metrics Using Higher Rating

Question	True Negative	False Positive	False Negative	True Positive	Sensitivity	Specificity	AUC
Q1	37	1	8	64	0.889	0.974	0.931
Q2	44	1	23	42	0.646	0.978	0.812
Q3	0	0	0	110	1	NA	NA
Q4	77	0	11	22	0.667	1	0.833
Q5	28	1	1	80	0.988	0.966	0.977
Q6	2	1	0	107	1	0.667	0.833
Q7	2	0	1	107	0.991	1	0.995
Q8	9	1	2	98	0.98	0.9	0.94
Q9	14	1	6	89	0.937	0.933	0.935
Q10	34	0	5	71	0.934	1	0.967
Q11	12	0	7	91	0.929	1	0.964
Q12	30	0	5	75	0.938	1	0.969
Q13	5	0	2	103	0.981	1	0.99
Q14	32	0	9	69	0.885	1	0.942
Q15	15	0	3	92	0.968	1	0.984
Q16	55	0	10	45	0.818	1	0.909
Q17	19	0	3	88	0.967	1	0.984
Q18	78	1	4	27	0.871	0.987	0.929
Q19	103	0	3	4	0.571	1	0.786
Q20	14	0	2	94	0.979	1	0.99
Q21	99	0	3	8	0.727	1	0.864
Q22	31	0	11	68	0.861	1	0.93
Q23	12	0	5	93	0.949	1	0.974
Q24	31	0	3	76	0.962	1	0.981
Q25	103	1	3	3	0.5	0.99	0.745
Q26	61	0	14	35	0.714	1	0.857
